# Enrollment in Dual-Eligible Special Needs Plans and Disenrollment Rates

**DOI:** 10.1001/jamahealthforum.2025.1748

**Published:** 2025-07-03

**Authors:** David J. Meyers, Eliza Macneal, Kendra Offiaeli, Eric T. Roberts

**Affiliations:** 1 Department of Health Services, Policy, and Practice, Brown University School of Public Health, Providence, Rhode Island; 2 Department of Medicine, Perelman School of Medicine, University of Pennsylvania, Philadelphia

## Abstract

**Importance:**

Medicare beneficiaries dually enrolled in Medicare and Medicaid have some of the highest care needs. Finding ways to support dually eligible beneficiaries in the Medicare Advantage (MA) program has become a policy goal.

**Objective:**

To determine if enrollment in different MA plan types is associated with differences in disenrollment.

**Design, Setting, and Participants:**

This cross-sectional study included Medicare enrollment data from dually eligible Medicare beneficiaries in 2021. Analyses were conducted between March 2024 and February 2025. Data were analyzed from January through March 2025.

**Exposure:**

Enrollment in different MA plan types, including those that exclusively serve dual-eligible beneficiaries (coordination-only, dual-eligible special needs plans [D-SNPs] and fully integrated D-SNPs [FIDE-SNPs]), standard MA plans that serve dual-eligible and non–dual-eligible beneficiaries, and D-SNP look-alike plans, defined as standard MA plans that primarily enroll dual-eligible beneficiaries.

**Main Outcomes and Measures:**

One-year disenrollment from one plan to another or to traditional Medicare.

**Results:**

Among 2 698 434 dually eligible beneficiaries in 2021, the mean (SD) age was 66.9 (14.1) years, and 62.5% were female individuals. Of dual-eligible beneficiaries enrolled in FIDE-SNPs in 2021, 19 001 (8.1%) disenrolled by 2022. Of those enrolled in coordination-only D-SNPs, D-SNP look-alikes, and standard MA plans in 2021, disenrollment rates were 18.3%, 30.5%, and 28.2%, respectively. Disenrollment rates were higher for Black beneficiaries and those who used more health services, including inpatient stays and more days of nursing home care.

**Conclusions and Relevance:**

The results of this cross-sectional study suggest that FIDE-SNPs retained their members at higher rates, which could be a sign of improved care experiences. Understanding how FIDE-SNPs may be affecting patient care will be important moving forward.

## Key Points

**Question:**

Is enrollment in Medicare Advantage plans with greater degrees of integration associated with lower plan disenrollment rates?

**Findings:**

In this cross-sectional study of 2.7 million dually eligible Medicare beneficiaries, dually eligible Medicare and Medicaid beneficiaries enrolled in fully integrated dual-eligible special needs plans had substantially lower disenrollment rates compared with beneficiaries enrolled in other Medicare Advantage plan types.

**Meaning:**

The study results suggest that fully integrated plans retained their members at higher rates, which could be a sign of improved care experiences.

## Teaser

This cross-sectional study examines if enrollment in different Medicare Advantage plan types is associated with differences in disenrollment.

## Introduction

In the US Medicare program, beneficiaries who are dually eligible for Medicare and Medicaid are among those with the most substantial health care needs. Dually eligible individuals represent 19% of Medicare beneficiaries, yet they account for 35% of Medicare costs, partially due to their greater burden of chronic disease and greater social needs.^1,2,3^ Over time, a growing share of dually eligible beneficiaries have been enrolling in Medicare Advantage (MA) plans, which are private plans paid by the US Centers for Medicare & Medicaid Services (CMS) to manage Medicare-covered services and spending for enrollees.^4,5,6^ Finding ways to support dually eligible beneficiaries in the MA program has become a policy goal.

One strategy intended to improve care for dually eligible Medicare beneficiaries has been the development of dually eligible special needs plans (D-SNPs). D-SNPs are MA plans that exclusively serve dually eligible beneficiaries and coordinate Medicare and Medicaid benefits.^7^ There is variation in how much D-SNPs coordinate care with Medicaid. For example, coordination-only D-SNPs coordinate some care and benefits with Medicaid, while fully integrated D-SNPs (FIDE-SNPs) bear risk for Medicare and Medicaid spending, promoting greater integration between the 2 programs.^8^ Research on the success of integrated care programs for dually eligible beneficiaries has yielded mixed findings, with a recent systematic review finding inconclusive evidence on whether integrated care plans, such as FIDE-SNPs, were associated with improved outcomes.^9^ As enrollment in MA continues to grow, further research is needed to understand the role different MA plan types play in addressing the care needs of dually eligible beneficiaries.

Disenrollment from an MA plan is often considered indicative of satisfaction with the plan and the quality of care it provides.^10,11,12,13,14,15^ While most MA beneficiaries are locked into a single plan all year, dually eligible beneficiaries can switch between MA plans or move to the traditional Medicare program on a monthly basis. Prior work has found that dually eligible beneficiaries tend to disenroll from their plans at more than double the rate of non–dually eligible beneficiaries.^16,17^ While disenrollment may indicate that a beneficiary is selecting a plan that is better aligned with their needs, disenrollment can also disrupt the continuity of care and signal poor quality or limited access to care in the plan a beneficiary chose to leave.^18^ Additionally, concerns exist that dual-eligible beneficiaries switching plans may enroll in other MA plans, such as D-SNP look-alikes plans, that primarily enroll dual-eligible beneficiaries but are not subject to D-SNP requirements to coordinate care.^19^

Despite the growth of D-SNPs in recent years, it is not currently known how disenrollment rates among dually eligible beneficiaries vary among these and other MA plan types. There is also limited evidence about the types of plans to which beneficiaries switch. In this study, we compared disenrollment rates for dually eligible beneficiaries across MA plan types, including D-SNPs, analyzed the characteristics of the plans beneficiaries switched to, and assessed whether disenrollment rates varied based on beneficiary characteristics.

## Methods

### MA Plan Type Classification

We analyzed person-level enrollment data among dual-eligible beneficiaries across 4 MA plan types: coordination-only D-SNPs, FIDE-SNPs, D-SNP look-alike plans, and standard MA plans. We defined standard MA plans as MA plans that did not serve specific populations, such as special needs plans (SNPs), Medicare-Medicaid plans (MMPs), or programs of all-inclusive care for elderly individuals (PACE). We defined D-SNP look-alike plans as otherwise standard MA plans in which dual-eligible individuals (including those with either full or partial Medicaid) represented more than half of plan enrollees.^20^

This study was approved by the University of Pennsylvania and Brown University institutional review boards as exempt research and received a waiver of informed consent because the study was a secondary analysis of deidentified data. The study followed the Strengthening the Reporting of Observational Studies in Epidemiology (STROBE) reporting guidelines.

### Data

The primary data source was the Medicare Master Beneficiary Summary File (MBSF) from 2018 to 2022, which we used to identify the study sample, track the MA plans of beneficiaries from one year to the next, and assess demographic characteristics. We linked the MBSF to CMS SNP reports to classify coordination-only D-SNPs and FIDE-SNPs; CMS Medicare Advantage plan reports to assess parent organizations for all plan types; and CMS part C crosswalk files to follow plans longitudinally, including plans with changes to the MA contract or plan identifiers (eg, due-to-plan consolidations). We used CMS reports of MA enrollment by plan and county to identify counties where plan availability was discontinued between years. In addition, we used the Medicare Provider Analysis and Review file and the Minimum Data Set from 2021 to measure beneficiaries’ annual number of inpatient hospital stays and number of days in a nursing facility, respectively.

### Study Sample

This study focused on beneficiaries with eligibility for full Medicaid. These individuals represent approximately three-quarters of all dual-eligible individuals, qualify for Medicaid-funded services (including long-term care), and are a primary focus of policy efforts to integrate care across Medicare and Medicaid.^3,21^ We analyzed annual cross-sectional samples of dual-eligible beneficiaries with full Medicaid and initial enrollment in an MA plan (coordination-only D-SNPs, FIDE-SNPs, D-SNP look-alike plans, or standard MA plans) in January from 2018 to 2021. To limit our analysis to beneficiaries who likely made an elective decision to stay in or switch from their Medicare plan, we excluded beneficiaries who died, lost full dual eligibility status, moved to another county, or were enrolled in an MA plan that was terminated during the window during which we examined disenrollment. We also excluded beneficiaries whose enrolled plan was terminated or otherwise stopped offering services in their residential county (plan discontinuation was inferred from the CMS database on whether enrollment in the plan-county declined to fewer than 50 individuals).

### Outcome Variables

We used monthly MA contract and plan benefit package identifiers in the MBSF to identify beneficiaries who disenrolled from their January MA plan in 1 year by January of the following year. A contract may include multiple plan benefit packages offered by the same parent organization in a market. We identified a plan as the combination of the contract and plan benefit package identifier in the MBSF. To account for changes in MA contract and plan identifiers, we linked each beneficiary to a persistent plan identifier, constructed from the CMS part C crosswalk file, which compared MA contract and plan benefit package identifiers across years. We identified disenrollees as beneficiaries who did not have the same persistent plan identifier between study years. Among disenrollees, we classified the MA plan type of beneficiaries as a coordination-only D-SNP, FIDE-SNP, D-SNP look-alike, standard MA plan, traditional Medicare, or other managed care plan (including institutional SNPs, chronic condition SNPs, MMPs, and PACE). In analyses using 2021 and 2022 data, we additionally examined disenrollment from and enrollment into highly integrated D-SNPs (HIDE-SNPs), an additional category of D-SNPs that CMS designated in 2021. HIDE-SNPs attain some financial integration with Medicaid because these plans bear risk for either Medicaid-funded long-term care or behavioral health spending.

### Analyses

We conducted 4 primary analyses. First, among annual cohorts of beneficiaries enrolled in MA plans from 2018 to 2021, we examined 1-year disenrollment rates by plan type (measured from 2019-2022). Next, among those who disenrolled from an MA plan between 2021 and 2022, we examined the types of plans they switched into, distinguishing between coordination-only D-SNPs, FIDE-SNPs, D-SNP look-alike plans, standard health maintenance organization or preferred provider organization MA plans not classified as D-SNPs, other integrated arrangements (eg, MMPs or PACE), or traditional Medicare. Third, among those who disenrolled between 2021 and 2022, we compared disenrollment rates across coordination-only D-SNPs, FIDE-SNPs, D-SNP look-alikes, and standard MA plans as stratified by age, sex, race and ethnicity, original reason for Medicare entitlement, census region, and number of hospitalizations and nursing facility days in 2021. Fourth, among disenrollees who in 2022 switched to a new plan of the same type as their 2021 plan, we examined the proportions who switched to a different MA plan offered by the same vs a different parent organization or within vs outside of the same MA contract. Analyses were conducted between March 2024 and February 2025 using SAS, version 9.4 (SAS Institute).

### Supplemental Analyses

We conducted 2 supplemental analyses. First, to examine disenrollment over a longer time window, we examined cumulative disenrollment during the 3-year period between 2018 and 2021. For this analysis, we excluded from our sample beneficiaries who died, lost full dual eligibility status, moved between counties, or were enrolled in an MA plan that was terminated between January 2018 and January 2021. Second, we estimated overall disenrollment rates, limiting only to states where FIDE-SNPs were available, to ensure that the results were not affected by states where FIDE-SNPs did not exist. To account for further state-level variation based on factors like the number and types of alternative plans available, we reported these disenrollment rates as within-state marginal means based on a linear regression at the beneficiary level modeling disenrollment based on plan type and state fixed effects.

## Results

### Sample Population

The study population included annual cross-sectional samples of dual-eligible beneficiaries with full Medicaid coverage enrolled in an MA plan. In 2021, this sample included 2 698 434 dual-eligible beneficiaries with full Medicaid coverage who were enrolled in a coordination-only D-SNP, FIDE-SNP, HIDE-SNP, D-SNP look-alike, or standard MA plan (eFigure 1 in Supplement 1). The mean (SD) age was 66.9 (14.1) years, and 62.5% were female individuals

### Disenrollment Rates Over Time and Trends by Plan Type

Among dual-eligible beneficiaries enrolled in FIDE-SNPs in 2021, 19 001 (8.1%) disenrolled by 2022 (Figure). Among those enrolled in coordination-only D-SNPs, D-SNP look-alikes, and standard MA plans in 2021, disenrollment rates were 18.3%, 30.5%, and 28.2%, respectively. Annual disenrollment rates were relatively similar across annual cohorts initially observed from 2018 to 2021 (disenrollment observed from 2019-2022). However, from 2020 to 2022, disenrollment rates among individuals in D-SNP look-alike plans rapidly increased from 19.1% to 30.5%.

**Figure.**
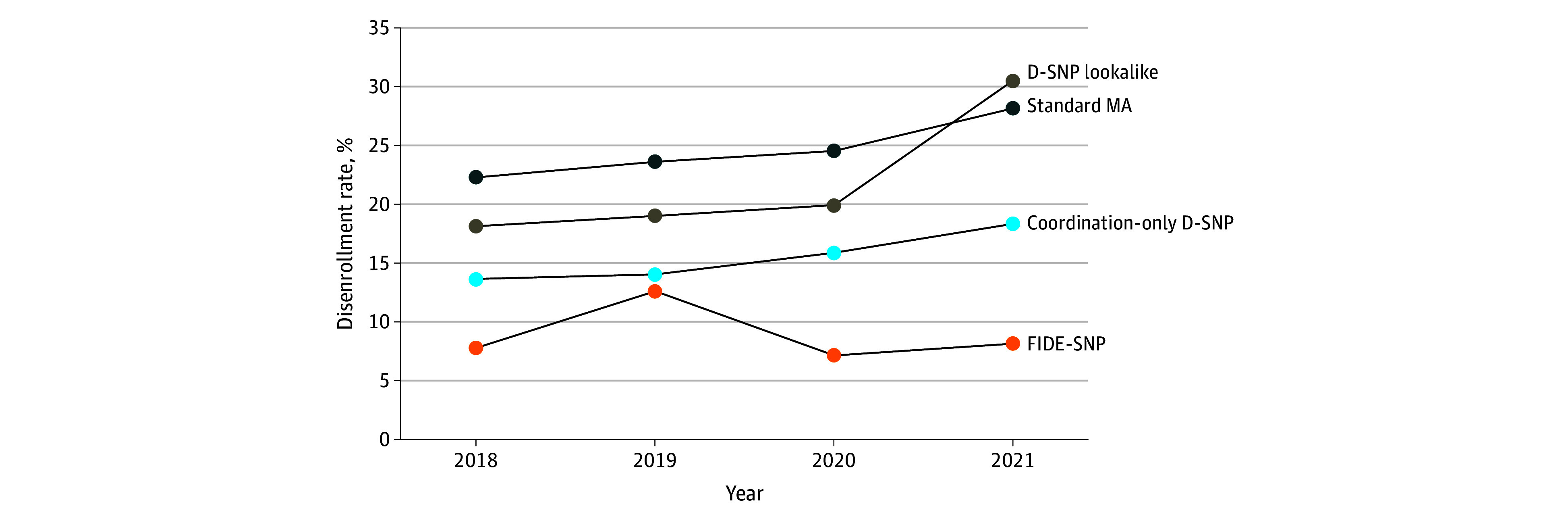
One-Year Disenrollment by Medicare Advantage (MA) Plan Type Over Time The analysis included annual cross-sectional cohorts of dual-eligible beneficiaries whose initial MA plan enrollment was measured in each January from 2018 to 2021. One-year disenrollment was measured in each cohort in each January from 2019 to 2022. Disenrollment from highly integrated dual-eligible special needs plans (HIDE-SNPs) between 2021 and 2022 is not reported in this figure (HIDE-SNPs were not available prior to 2021). Beneficiaries who died, lost full dual eligibility status, or moved counties between years were excluded from the sample. Beneficiaries enrolled in an integrated special needs plan (SNP), coordination-only SNP, Medicare-Medicaid plans (MMPs), programs of all-inclusive care for elderly individuals (PACE), or a plan that was terminated or pulled from their county of residence are excluded from the sample. Percentages indicate the proportion of full-dual eligible enrollees in January of the given year who disenrolled by the following January. Beneficiaries switching plans as part of a plan consolidation are not considered to have disenrolled. Dual-eligible special needs plan (D-SNP) look-alikes were defined as MA plans other than SNPs, MMPs, or PACE plans for which full or partial dual-eligible individuals constituted more than half of enrollees in January of the given year. Standard MA plans were defined as MA plans that are not SNPs, MMPs, PACE plans, or D-SNP look-alikes. Disenrollment rates may have varied over time due to changes in the plans offered, regions where plans are offered, and enrollee mix within plans. The introduction of the HIDE-SNP designation in 2021 yielded a drop in the number of coordination-only D-SNPs. One D-SNP look-alike was dropped from the analysis in 2021 due to an implausible disenrollment rate. The plotted point reflects the exclusion of this plan. FIDE-SNP indicates fully integrated D-SNPs.

The largest proportion of dual-eligible beneficiaries disenrolling from FIDE-SNPs in 2021 switched into an alternative FIDE-SNP in 2022 (7932 [41.8%]) (Table 1), while those disenrolling from standard MA plans primarily switched into coordination-only D-SNPs (50 994 [32.4%]). The largest proportions of dual-eligible individuals disenrolling from coordination-only D-SNPs switched into other coordination-only D-SNPs (122 370 [62.1%]), and the largest share from D-SNP look-alike plans also switched into coordination-only D-SNPs (20 154 [32.7%]). Among individuals who disenrolled from their plan in 2021, enrollees in FIDE-SNPs had the highest likelihood of switching to traditional Medicare (3084 [16.2%]).

**Table 1.** Type of Medicare Enrollment in 2022 Among Full-Benefit, Dual-Eligible Individuals Enrolled in Medicare Advantage (MA) in 2021

MA plan in 2021^a^	No. (%)		Medicare plan type in 2022 among disenrollees, No. (% of disenrollees)						
	Stayed enrolled^b^	Disenrolled	Coordination-only D-SNP	HIDE-SNP	FIDE-SNP	D-SNP look-alike	Standard MA^c^	Traditional Medicare	Other plan^d^
Coordination-only D-SNP	877 779 (81.7)	196 966 (18.3)	122 370 (62.1)	30 918 (15.7)	4689 (2.4	2539 (1.3)	9516 (4.8)	18 035 (9.12)	8899 (4.5)
HIDE-SNP	515 608 (82.1)	112 674 (17.9)	12 031 (10.7)	73 405 (65.2)	9753 (8.7)	2141 (1.9)	4748 (4.2)	8340 (7.4)	2256 (2.0)
FIDE-SNP	215 667 (91.9)	19 001 (8.1)	2623 (13.8)	1162 (6.1)	7932 (41.8)	1373 (7.2)	2078 (10.9)	3084 (16.2)	749 (3.9)
D-SNP lookalike^e^	140 146 (69.5)	61 549 (30.5)	20 154 (32.7)	7692 (12.5)	1532 (2.5)	11 139 (18.1)	10 038 (16.3)	6365 (10.3)	4629 (7.5)
Standard MA^f^	401 692 (71.9)	157 352 (28.2)	50 994 (32.4)	20 987 (13.3)	7403 (4.7)	14 124 (9.0)	31 796 (20.2)	17 819 (11.3)	14 229 (9.0)
Total	2 150 892 (79.7)	547 542 (20.3)	208 182 (36.4)	157 963 (27.6)	31 309 (5.5)	31 649 (5.5)	58 310 (10.2)	54 093 (9.5)	30 834 (5.4)

Abbreviations: C-SNP, coordination-only special needs plan; D-SNP, dual-eligible special needs plan; FIDE-SNP, fully integrated dual-eligible special needs plan; HIDE-SNP, highly integrated dual-eligible special needs plan; MMP, Medicare-Medicaid plan; PACE, programs of all-inclusive care for elderly individuals; SNP, special needs plan.

^a^ Analysis included dual-eligible beneficiaries whose initial MA plan enrollment was measured in January 2021, and disenrollment was measured by January 2022.

^b^ Beneficiaries changing plans as a result of plan consolidation were considered to have stayed enrolled.

^c^ Beneficiaries who died, lost full dual eligibility status, or moved counties between years were excluded from the sample. Beneficiaries enrolled in an integrated SNP, C-SNP, MMPs, PACE, or a plan that was terminated or pulled from their county of residence are excluded from the sample.

^d^ Other plans included integrated SNPs, C-SNPs, MMPs, and PACE plans.

^e^ D-SNP look-alikes were defined as MA plans other than SNPs, MMPs, or PACE plans for which full or partial duals constitute more than half of enrollees.

^f^ Standard MA plans were defined as MA plans that were not SNPs, MMPs, PACE plans, or D-SNP lookalikes.

### Disenrollment Among Subpopulations by Plan Type

Across all plan types, disenrollment rates between 2021 and 2022 were higher among beneficiaries aged 65 to 72 years (197 543 [23.1%]) vs those younger than 65 years (178 271 [19.8%]) or older than 72 years (171 728 [18.2%]) (Table 2). Disenrollment rates were highest among Black beneficiaries across all SNP types (152 830 [24.5%]) compared with individuals of other racial and ethnic groups.

**Table 2.** Characteristics of Beneficiaries Who Disenrolled From Different Plan Types

Population	No. (%) of population that disenrolled^a^					
	All plans (N = 2 698 434)^b^	2021 Plan type				
		FIDE (n = 234 668)	HIDE (n = 628 282)	Coordination-only D-SNP(n = 1 074 745)	D-SNP look-alike (n = 201 695)^c^	Standard MA (n = 559 044)^d^
Overall	547 542 (20.3)	19 001 (8.1)	112 674 (17.9)	196 966 (18.3)	61 549 (30.5)	157 352 (28.2)
Age, y						
<65	178 271 (19.8)	4199 (8.7)	33 027 (16.8)	76 306 (17.3)	13 940 (30.2)	50 799 (29.9)
65-72	197 543 (23.1)	6365 (8.9)	39 396 (19.6)	71 558 (21.3)	23 543 (32.1)	56 681 (32.9)
≥73	171 728 (18.2)	8437 (7.3)	40 251 (17.5)	49 102 (16.5)	24 066 (29.3)	49 872 (23.0)
Sex						
Female	336 752 (20.0)	11 545 (7.6)	70 830 (18.0)	121 946 (18.1)	36 732 (30.6)	95 699 (27.8)
Male	210 790 (20.8)	7456 (9.0)	41 844 (17.8)	75 020 (18.8)	24 817 (30.4)	61 653 (28.7)
Race and ethnicity						
Asian/Pacific Islander	45 595 (18.1)	2038 (8.5)	11 286 (16.3)	13 931 (15.6)	10 962 (29.8)	7378 (22.6)
Black	152 830 (24.5)	4064 (11.0)	25 264 (22.1)	72 573 (21.6)	10 077 (39.5)	40 852 (36.9
Hispanic	128 513 (19.4)	6224 (9.3)	41 866 (17.8)	28 003 (17.8)	24 840 (28.3)	27 580 (24.1)
White	208 516 (19.1)	6125 (6.3)	31 639 (16.7)	78 617 (16.8)	14 235 (30.1)	77 900 (27.0)
Other	12 088 (17.4)	550 (6.2)	2619 (13.4)	3842 (16.5)	1435 (32.8)	3642 (27.5)
Original reason for entitlement						
OASI	271 194 (20.0)	11 040 (7.9)	62 843 (18.1)	82 866 (18.5	37 927 (30.0)	76 518 (25.8)
DIB	272 656 (20.6)	7874 (8.4)	49 057 (17.7)	112 496 (18.1	23 375 (31.3)	79 854 (30.8)
ESKD	3692 (25.8)	87 (10.9)	774 (22.0)	1604 (24.3	247 (40.6)	980 (35.1)
Census region						
Northeast	120 852 (17.0)	14 438 (8.7)	16 380 (14.3)	60 052 (18.6)	2422 (30.5)	27 560 (27.5
Midwest	99 379 (23.0)	845 (2.3)	8767 (14.4)	40 028 (21.6)	6097 (32.6)	43 642 (33.0)
South	210 286 (24.5)	419 (16.1)	71 837 (24.9)	81 700 (19.4)	12 697 (50.4)	43 633 (35.6)
West	116 855 (16.8)	3295 (10.7)	15 661 (9.5)	15 150 (10.4)	40 313 (26.9)	42 436 (20.8)
No. of hospitalizations in 2021						
0	444 958 (19.6)	14 961 (7.7)	91 418 (17.1)	159 094 (17.6)	52 711 (30.0)	126 774 (27.2)
1	63 987 (22.9)	2419 (9.4)	13 255 (21.0)	23 497 (21.0)	5750.0 (32.6)	19 066 (31.5)
2	20 942 (25.4)	822 (10.3)	4203 (23.0)	7740 (23.5)	1758 (36.6)	6419 (34.9)
≥3	17 655 (28.6)	799 (13.9)	3798 (27.9)	6635 (26.1)	1330 (40.1)	5093 (37.5)
Days in a nursing facility in 2021						
0	500 831 (19.9)	16 770 (7.9)	105 375 (17.5)	183 148 (17.9)	58 733 (30.3)	136 805 (28.5)
1-30	12 658 (24.2)	575 (9.6)	2449 (23.2)	4498 (22.2)	1139 (35.1)	3997 (32.4)
31-99	8580 (30.7)	473 (13.5)	1694 (31.1)	2853 (29.0)	662 (41.9)	2898 (38.3)
≥100	25 473 (24.4)	1183 (9.3)	3156 (35.4)	6467 (30.9)	1015 (35.1)	13 652 (23.2)

Abbreviations: D-SNP, dual-eligible special needs plan; DIB, disability insurance benefit; ESKD, end-stage kidney disease; FIDE-SNP, fully integrated dual-eligible special needs plan; HIDE-SNP, highly integrated dual-eligible special needs plan; MA, Medicare Advantage; MMP, Medicare-Medicaid plan; OASI, old-age and survivors insurance; PACE, programs of all-inclusive care for elderly individuals; SNP, special needs plan.

^a^ Beneficiaries changing plans as a result of plan consolidation were not considered to have disenrolled.

^b^ Percentages reflect the proportion of beneficiaries of the given plan type and subpopulation who disenrolled. For example, 8.7% of full-benefit, dual-eligible individuals younger than 65 years who were enrolled in a FIDE-SNP in January 2021 disenrolled from their plan by January 2022.

^c^ D-SNP look-alikes were defined as MA plans other than SNPs, MMPs, or PACE plans for which full or partial duals constitute more than half of enrollees. One D-SNP lookalike was dropped from the analysis in 2021 due to an implausible disenrollment rate.

^d^ Standard MA plans were defined as MA plans that are not SNPs, MMPs, PACE plans, or D-SNP look-alikes.

Across all plan types, disenrollment rates between 2021 and 2022 were greater among beneficiaries with more hospitalizations in 2021 (eg, a disenrollment rate of 28.6% [n = 17 655] among those with 3 or more hospitalizations vs 19.6% [n = 444 958] among those with no hospitalizations in 2021). However, disenrollment from FIDE-SNPs among beneficiaries with 3 or more hospitalizations in 2021 (799 [13.9%]) was still lower than that among beneficiaries with no hospitalizations in all other plan types. Across all plan types, disenrollment rates were also generally greater among beneficiaries with more days spent in a nursing facility in 2021. However, beneficiaries with 100 or more days in nursing facilities in 2021 disenrolled at a somewhat lower rate between 2021 and 2022 (25 473 [24.4%]) compared with those with 31 to 99 days (8580 [30.7%]). Enrollees in FIDE-SNPs with 100 or more days of nursing home care in 2021 disenrolled from these plans at a rate of 9.3% between 2021 and 2022.

### Disenrollment Within Contracts and Parent Organizations

Among beneficiaries who switched to a plan of the same type as their 2021 plan, we assessed rates of disenrollment and switching to new plan of benefit packages within vs between contracts and parent organizations. Among dual-eligible individuals who switched from one FIDE-SNP to another between 2021 and 2022, 721 (9.1%) switched to a new plan benefit package within the same contract, whereas 726 (9.2%) switched to a different FIDE-SNP offered by the same parent organization as their original plan (Table 3). Therefore, 90.9% left their original contract, and 90.8% left their original parent organization. Among dual-eligible individuals switching from one coordination-only D-SNP to another and from one D-SNP look-alike to another, 94.7% and 97.2% left their original contract, respectively, and 81.8% and 89.1% left their original parent organization, respectively.

**Table 3.** Proportion of Within-Contract and Within-Organization Churn Among Full-Benefit, Dual-Eligible Individuals Who Switched From One Medicare Advantage Plan to Another of the Same Type Between 2021 and 2022

Plan type in 2021 and 2022^a^	No.	Disenrolled, No. (%)	
		Within contract	Within parent organization
FIDE-SNP	7932	721 (9.1)	726 (9.2)
Coordination-only D-SNP	122 370	6399 (5.2)	22 267 (18.2)
D-SNP lookalike^b^	11 472	327 (2.9)	1247 (10.9)

Abbreviations: D-SNP, dual-eligible special needs plan; FIDE-SNP, fully integrated dual-eligible special needs plan.

^a^ Beneficiaries enrolled in a plan that was terminated or pulled from their county of residence were excluded from the sample. Beneficiaries who changed plans as a result of plan consolidation were not considered to have disenrolled.

^b^ D-SNP look-alikes were defined as Medicare Advantage plans other than special needs plans, Medicare-Medicaid plans, or programs of all-inclusive care for elderly individuals for which full or partially dual-eligible individuals constituted more than half of enrollees.

### Supplemental Analyses

In our first supplementary analysis, we analyzed 1 267 077 dual-eligible beneficiaries who were enrolled in a D-SNP, D-SNP look-alike, or standard MA plan in January 2018 and remained alive and enrolled in full Medicaid coverage by January 2021 (excluding those who moved counties or whose MA plan was no longer available during this period). During this 3-year period, disenrollment rates across FIDE-SNPs, coordination-only D-SNPs, D-SNP look-alikes, and standard MA plans were 20.7%, 29.0%, 36.2%, and 44.4%, respectively (eTable 1 in Supplement 1). Among those who disenrolled from their MA plan by January 2018, the proportions switching to traditional Medicare or another MA plan in the same or different category by January 2021 were mostly similar to our main analyses. However, one change in 2021 was the introduction of HIDE-SNPs, and some dual-eligible beneficiaries switched to these plans by that year. For example, among beneficiaries who disenrolled from coordination-only D-SNPs between 2018 and 2021, 31.8% switched to a HIDE-SNP, while among those who disenrolled from FIDE-SNPs, 2.8% switched to a HIDE-SNP. In our secondary supplementary analysis, we found that after limiting our sample to states that had at least 1 FIDE-SNP and examining within-state disenrollment, we found very similar results over time in disenrollment trends (eFigure 2 in Supplement 1).

## Discussion

This cross-sectional, national study of MA plan disenrollment among dually eligible Medicare beneficiaries had 2 key findings. First, we found that dual-eligible beneficiaries initially enrolled in FIDE-SNPs had substantially lower disenrollment rates than beneficiaries in other types of D-SNP plans, while dual-eligible beneficiaries initially enrolled in standard MA plans had the highest rates of disenrollment. These patterns remained consistent in analyses of annual disenrollment rates among cohorts initially enrolled in MA plans between 2018 and 2021. Second, disenrollment rates were higher among Black beneficiaries and those with greater prior use of hospital and nursing home care. In additional analyses, we found that most disenrollments were to different companies than the one the beneficiary originated in, and that disenrollment rates were higher using a longer follow-up window.

Prior work documented variations in disenrollment rates among dual-eligible beneficiaries enrolled in MA contracts composed primarily of D-SNPs but did not find consistent associations between contract characteristics and disenrollment rates.^22^ In this study, we examined disenrollment rates at the more granular level of the plan, enabling us to differentiate between D-SNPs with different levels of Medicaid integration. We found that dual-eligible beneficiaries enrolled in FIDE-SNPs had much lower rates of disenrollment compared with beneficiaries in other plan types. Several factors may have contributed to this finding. First, most dual-eligible beneficiaries were enrolled in Medicaid managed care plans in addition to their MA plan. In FIDE-SNPs, the same parent company must run the Medicaid and Medicare plan, which may provide a more seamless experience for beneficiaries. Conversely, beneficiaries in other plan types may not experience the same type of integration and have less reason to remain enrolled in the same plan. Second, it could be the case that FIDE-SNPs provide better experiences for beneficiaries, leading to fewer beneficiaries wanting to leave their plan. For example, a recent study found that enrollees in FIDE-SNPs reported higher overall ratings of their plans than enrollees in coordination-only D-SNPs and standard MA plans, although ratings differed in other domains of patient-reported experiences.^23^ Third, CMS has used several default enrollment strategies to guide beneficiaries into FIDE-SNPs.^21^ While we could not assess in this study if any disenrollment was associated with these default enrollments or intentional nudges by CMS, it may help to explain why disenrollment rates were smaller among FIDE-SNPs compared with other plan types.

Our analysis revealed that beneficiaries with higher baseline use of inpatient and nursing home care were more likely to disenroll from MA plans than beneficiaries with lower utilization. This aligned with prior research that found higher disenrollment rates from MA contracts among beneficiaries with greater care needs.^17^ Although we found that dual-eligible beneficiaries with greater baseline utilization were also more likely to leave FIDE-SNPs than those with lower utilization, overall disenrollment rates from FIDE-SNPs across high- and low-utilizing populations remained lower than those of other plan types.

Another finding was that Black and Hispanic dual-eligible beneficiaries tended to disenroll from plans at higher rates across all plan types. This aligned with prior findings that Black and Hispanic beneficiaries across the MA program tend to disenroll at higher rates.^24,25,26,27^ Prior qualitative work has suggested this may be partially due to beneficiaries of racial and ethnic minority groups being more often guided to plans by brokers, who may not ultimately know which plan is best for their specific care needs.^28^ Compared with other plan types, FIDE-SNPs had relatively lower disenrollment rates among Black and Hispanic beneficiaries. However, disenrollment rates from FIDE-SNPs were still higher for Black and Hispanic beneficiaries compared with White beneficiaries. More work is needed to understand whether specific plan characteristics contribute to such disenrollment patterns across beneficiary subgroups and plan types.

### Limitations

Our study had several limitations. First, this was an associational analysis, and our comparisons do not have a causal interpretation. Second, our primary analyses examined annual disenrollment (ie, from one year to the next). However, we found that overall disenrollment rates remained relatively stable over time, and our primary results were maintained in a subsample of beneficiaries followed up over 3 years. Third, we could not assess the reasons why beneficiaries disenrolled from plans. Although CMS surveys beneficiaries about reasons for MA plan disenrollment, these data are only publicly reported at the contract level, and MA contracts often comprise multiple plans. Therefore, these data were not sufficiently granular to examine beneficiary-reported reasons for disenrollment from specific MA plan types. Despite these limitations, to our knowledge, this is the first national study to granularly compare disenrollment rates across MA plan types, including D-SNPs attaining different levels of Medicaid integration.

## Conclusions

This cross-sectional study of dual-eligible beneficiaries found that disenrollment rates from D-SNPs were lower among beneficiaries enrolled in FIDE-SNPs compared with other plan types within the MA program. However, across all plan types, disenrollment rates were relatively higher among Black beneficiaries and individuals with greater hospital and nursing home use. Although we could not determine what was associated with FIDE-SNPs having relatively lower disenrollment rates than other plans, these results may indicate some benefit from the improved coordination and integration these plans offer for beneficiary experiences. These results also underscore a need for further research to assess potential reasons for variations in disenrollment rates across subpopulations of dual-eligible beneficiaries and different MA plan types.

## Notes

Supplement 1. eFigure 1. Flowchart of analytical sample construction eTable 1. Type of Medicare enrollment in 2021 among full benefit dual-eligibles enrolled in D-SNPs, D-SNP lookalikes, and standard MA plans in 2018 eTable 2. Reported reasons for Medicare Advantage disenrollment in 2019 by plan type eFigure 2. Within-state proportion of full benefit dual-eligibles residing in states with FIDE-SNPs who disenrolled from their Medicare Advantage plan, 2018-2021

Supplement 2. Data sharing statement
