# Supplementary material for: Enrollment in Dual-Eligible Special Needs Plans and Disenrollment Rates
Source: JAMA Health Forum. 2025 Jul 3;6(7):e251748. doi: 10.1001/jamahealthforum.2025.1748 (PMC12232179; doi:10.1001/jamahealthforum.2025.1748)
Supplement: Supplement 1. — eFigure 1. Flowchart of analytical sample construction eTable 1. Type of Medicare enrollment in 2021 among full benefit dual-eligibles enrolled in D-SNPs, D-SNP lookalikes, and standard MA plans in 2018 eTable 2. Reported reasons for Medicare Advantage disenrollment in 2019 by plan type eFigure 2. Within-state proportion of full benefit dual-eligibles residing in states with FIDE-SNPs who disenrolled from their Medicare Advantage plan, 2018-2021 [file jamahealthforum-e251748-s001.pdf]

Supplemental Online Content

Meyers DJ, Macneal E, Offiaeli K, Roberts ET. Enrollment in dual-eligible special needs plans and disenrollment rates. *JAMA Health Forum*. Published online July 3. 2025. doi:10.1001/jamahealthforum.2025.1748

eFigure 1. Flowchart of analytical sample construction
eTable 1. Type of Medicare enrollment in 2021 among full benefit dual-eligibles enrolled in D-SNPs, D-SNP lookalikes, and standard MA plans in 2018
eTable 2. Reported reasons for Medicare Advantage disenrollment in 2019 by plan type
eFigure 2. Within-state proportion of full benefit dual-eligibles residing in states with FIDE-SNPs who disenrolled from their Medicare Advantage plan, 2018-2021

This supplemental material has been provided by the authors to give readers additional information about their work.

© 2025 Meyers DJ et al. *JAMA Health Forum*.

eFigure 1. Flowchart of analytical sample construction

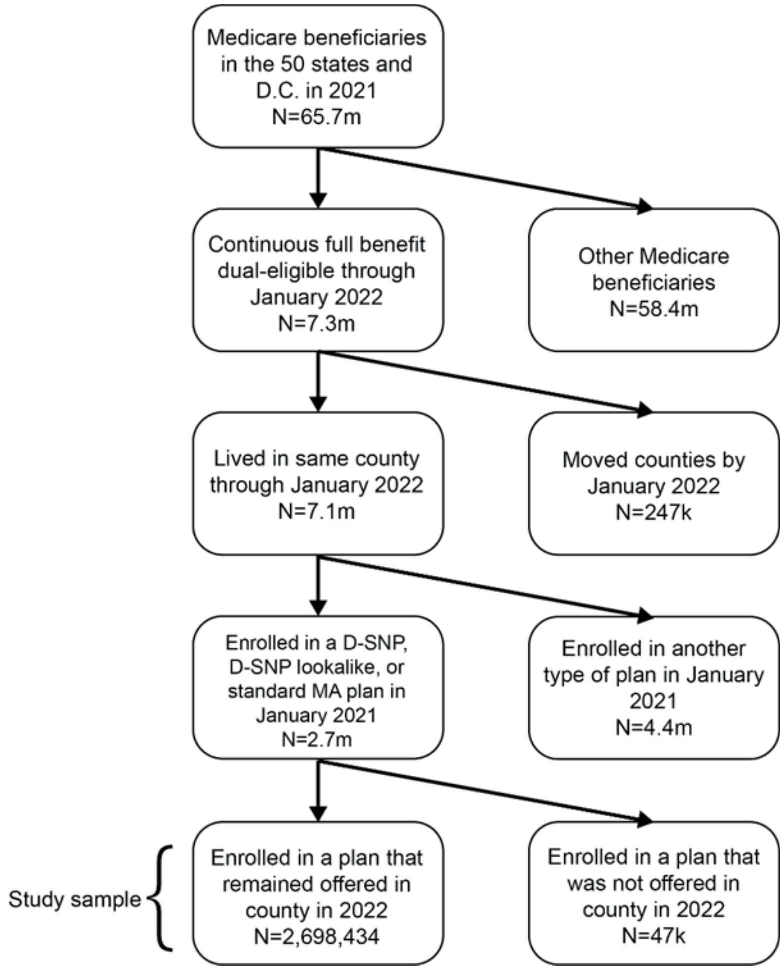

Notes: D-SNP lookalikes are defined as MA plans other than SNPs, MMPs, or PACE plans for which full or partial duals constitute more than half of enrollees in January of the given year. Standard MA plans are defined as MA plans that are not SNPs, MMPs, PACE plans, or D-SNP lookalikes.

Source: MBSF 2021-2022, CMS SNP reports 2021-2022, CMS MA enrollment reports by plan and county 2021-2022

© 2025 Meyers DJ et al. *JAMA Health Forum*.

eTable 1. Type of Medicare enrollment in 2021 among full benefit dual-eligibles enrolled in D-SNPs, D-SNP lookalikes, and standard MA plans in 2018

Population: Full dual-eligibles enrolled in a D-SNP, D-SNP lookalike, or standard MA plan in 2018 (a)

	**Stayed Enrolled** (b)		**Disenrolled**		**Medicare plan type in 2021 among disenrollees**					
					Coordination only D-SNP		HIDE-SNP (e)		FIDE-SNP	
**MA plan in 2018**	N	%	N	%	N	% of disenrollees	N	% of disenrollees	N	% of disenrollees
Coordination only D-SNP	577,323	71.05%	235,274	28.95%	84,803	36.04%	74,786	31.79%	12,285	5.22%
FIDE-SNP	84,486	79.32%	22,024	20.68%	463	2.10%	617	2.80%	13,193	59.90%
D-SNP lookalike (c)	69,545	63.80%	39,467	36.20%	2,314	5.86%	3,837	9.72%	1,209	3.06%
Standard MA (d)	132,838	55.59%	106,120	44.41%	35,740	33.68%	13,545	12.76%	5,082	4.79%
**Total**	864,192	68.20%	402,885	31.80%	123,320	30.61%	92,785	22.04%	31,769	7.55%

	**Medicare plan type in 2021 among disenrollees**							
	D-SNP lookalike		Standard MA		Traditional Medicare		Other plan (f)	
**MA plan in 2018**	N	% of disenrollees	N	% of disenrollees	N	% of disenrollees	N	% of disenrollees
Coordination only D-SNP	11,319	4.81%	11,770	5.00%	28,457	12.10%	11,854	5.04%
FIDE-SNP	1,175	5.34%	1,522	6.91%	4,045	18.37%	1,009	4.58%
D-SNP lookalike (c)	15,197	38.51%	4,981	12.62%	7,483	18.96%	4,446	11.27%
Standard MA (d)	9,787	9.22%	17,327	16.33%	15,444	14.55%	9,195	8.66%
**Total**	37,478	9.30%	35,600	8.84%	55,429	13.76%	26,504	6.58%

(a) Beneficiaries who died, lost full dual status, or moved counties between years are excluded from the sample. Beneficiaries enrolled in an I-SNP, C-SNP, MMP, PACE, or a plan that was terminated or pulled from their county of residence are excluded from the sample.
(b) Beneficiaries changing plans as a result of plan consolidation are considered to have stayed enrolled.
(c) D-SNP lookalikes are defined as MA plans other than SNPs, MMPs, or PACE plans for which full or partial duals constitute more than half of enrollees.

© 2025 Meyers DJ et al. *JAMA Health Forum*.

(d) Standard MA plans are defined as MA plans that are not SNPs, MMPs, PACE plans, or D-SNP lookalikes.
(e) The HIDE-SNP designation began in 2021.
(f) Other plans include I-SNPs, C-SNPs, MMPs, and PACE plans.
Notes: Beneficiaries switching plans as part of a plan consolidation or bifurcation are classified as staying enrolled. "Other plans" include I-SNPs, C-SNPs, MMPs, and PACE plans. Based on January enrollment in 2018 and 2021.
Source: 100% MBSF 2018-2021, CMS SNP data 2018-2021, CMS Parts C&D crosswalk files 2018-2021

© 2025 Meyers DJ et al. *JAMA Health Forum*.

eTable 2. Reported Reasons for Medicare Advantage disenrollment in 2019 by plan type

Population: 2019 MA contracts aggregated by plan type

		Disenrollment Reason									
		Problems getting needed care, coverage, and cost information		Problems with coverage of doctors and hospitals		Financial reasons for disenrollment		Problems with prescription drug benefits and coverage		Problems getting information and help from the plan	
**Plan type**	N total contracts	N response contracts	% indicated reason	N response contracts	% indicated reason	N response contracts	% indicated reason	N response contracts	% indicated reason	N response contracts	% indicated reason
FIDE-SNP	37	21	13.4%	21	14.4%	21	19.6%	21	33.2%	10	10.1%
Coordination Only D-SNP	155	123	18.4%	117	12.2%	121	18.2%	123	27.5%	63	10.1%
D-SNP lookalike	41	36	16.0%	36	15.8%	36	21.6%	36	31.5%	17	9.9%

Notes: Rates of disenrollment reasons are reported by contract, and contracts can comprise multiple plan types. To construct plan type averages, we weighted each contract by its share of enrollees in the plan type (among contracts contributing disenrollment reason data). For example, for 3 contracts (A, B, and C) with 50, 100, and 150 enrollees in FIDE-SNPs, respectively, we would give weights of 16.7% to contract A, 33.3% to contract B, and 50% to contract C when calculating the FIDE-SNP disenrollment reason rates.

Source: CMS MA & SNP enrollment files 2019, CMS Disenrollment Reasons Survey (DRS) contract report 2019

© 2025 Meyers DJ et al. *JAMA Health Forum*.

eFigure 2. Within-state proportion of full benefit dual-eligibles residing in states with FIDE-SNPs who disenrolled from their Medicare Advantage plan, 2018-2021

Population: Full dual-eligibles in Medicare Advantage (a) residing in states with at least one FIDE-SNP offered (b)

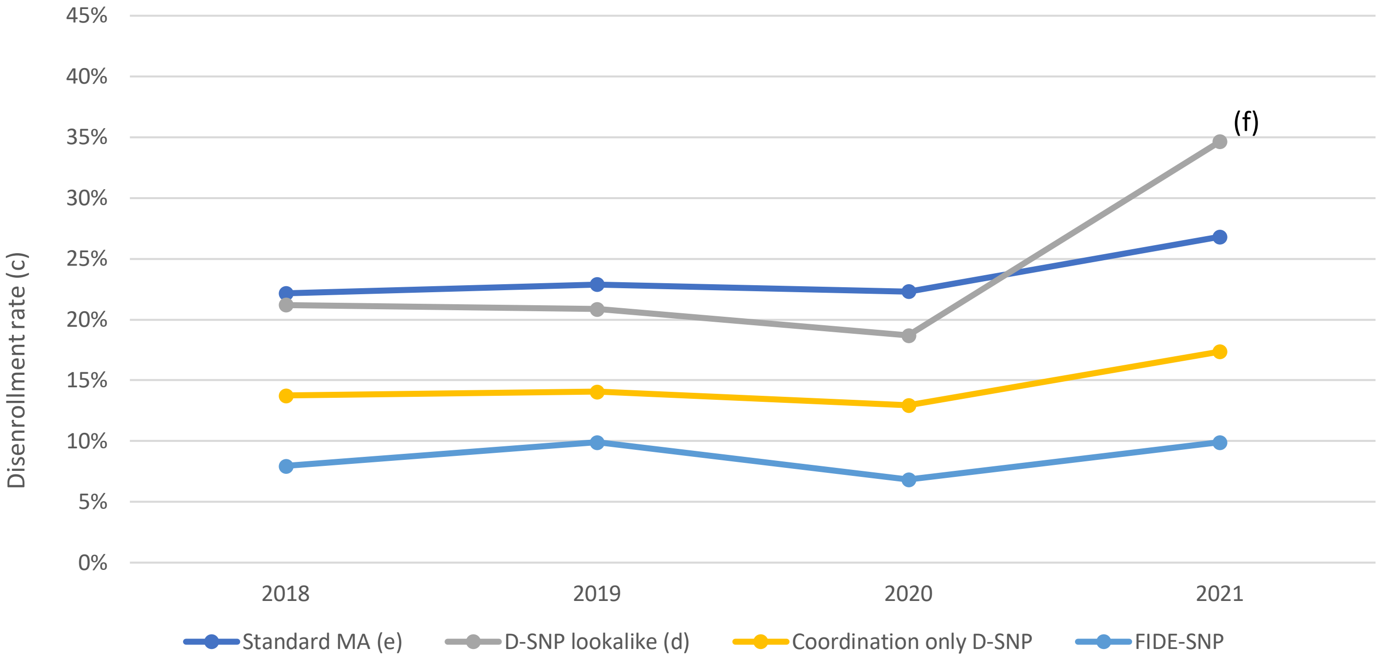

(a) Beneficiaries who died, lost full dual status, or moved counties between years are excluded from the sample. Beneficiaries enrolled in MMPs, I-SNPs, C-SNPs, PACE, or a plan that was terminated or pulled from their county of residence are excluded from the sample.
(b) AZ, CA, FL, ID, MA, MN, NJ, NY, TN, and WI offered FIDE-SNPs in all data years. PA offered FIDE-SNPs starting in 2020, and VA starting in 2021.
(c) Percentages indicate the within-state marginal mean proportion of full-dual eligible enrollees in January of the given year who disenrolled by the following January. Proportions are calculated from a linear regression of disenrollment based on plan type and state fixed effects. Beneficiaries switching plans as part of a plan consolidation are not considered to have disenrolled.
(d) D-SNP lookalikes are defined as MA plans other than SNPs, MMPs, or PACE plans for which full or partial duals constitute more than half of enrollees in January of the given year.
(d) Standard MA plans are defined as MA plans that are not SNPs, MMPs, PACE plans, or D-SNP lookalikes.
(e) Disenrollment rates may vary over time due to changes in plans offered, regions where plans are offered, and enrollee mix within plans. The introduction of the HIDE-SNP designation in 2021 yielded a drop in the number of Coordination Only D-SNPs.
(f) One D-SNP lookalike was dropped from the analysis in 2021 due to an implausible disenrollment rate.

Source: MBSF 2018-2022, CMS SNP reports 2018-2022, CMS MA enrollment reports by plan and county 2018-2022, CMS Parts C&D crosswalk files 2018-2022

© 2025 Meyers DJ et al. *JAMA Health Forum*.

© 2025 Meyers DJ et al. *JAMA Health Forum*.
