# Supplementary material for: Enrollment in Dual-Eligible Special Needs Plans and Disenrollment Rates
Source: JAMA Health Forum. 2025 Jul 3;6(7):e251748. doi: 10.1001/jamahealthforum.2025.1748 (PMC12232179; doi:10.1001/jamahealthforum.2025.1748)
Supplement: Supplement 2. — Data sharing statement [file jamahealthforum-e251748-s002.pdf]

Data Sharing Statement

Meyers. Enrollment in Dual-Eligible Special Needs Plans and Disenrollment Rates. *JAMA Health Forum*. Published July 03, 2025. doi:10.1001/jamahealthforum.2025.1748

Data
Data available: No

Additional Information
Explanation for why data not available: These data are only available under a DUA with CMS, however the authors can provide deidentified code upon request.
